# Multiplexed TrAEL-seq captures DNA replication dynamics in mammalian cells

**DOI:** 10.1093/nar/gkag212

**Published:** 2026-03-14

**Authors:** Neesha Kara, Laura Biggins, Alex Whale, Kieron May, Vera Grinkevich, Paola Garran-Garcia, Jhanavi Srinivasan, Peter J Rugg-Gunn, Claudia Ribeiro de Almeida, Samantha J Walker, Gabriele Picco, Mathew J Garnett, Simon Andrews, Aled Parry, Helen M R Robinson, Jonathan Houseley

**Affiliations:** Epigenetics programme, Babraham Institute, CB22 3AT Babraham, United Kingdom; Babraham Bioinformatics, Babraham Institute, CB22 3AT Babraham, United Kingdom; Epigenetics programme, Babraham Institute, CB22 3AT Babraham, United Kingdom; Epigenetics programme, Babraham Institute, CB22 3AT Babraham, United Kingdom; Artios Pharma, Babraham Research Campus, CB22 3AT Babraham, United Kingdom; Epigenetics programme, Babraham Institute, CB22 3AT Babraham, United Kingdom; Epigenetics programme, Babraham Institute, CB22 3AT Babraham, United Kingdom; Epigenetics programme, Babraham Institute, CB22 3AT Babraham, United Kingdom; Cambridge Stem Cell Institute, Jeffrey Cheah Biomedical Centre, University of Cambridge, CB2 0AW Cambridge, United Kingdom; Loke Centre for Trophoblast Research, University of Cambridge, CB2 3EG Cambridge, United Kingdom; Immunology Programme, Babraham Institute, CB22 3AT Babraham, United Kingdom; Somatic Genomics Programme, Wellcome Sanger Institute, Cambridge, United Kingdom; Somatic Genomics Programme, Wellcome Sanger Institute, Cambridge, United Kingdom; Somatic Genomics Programme, Wellcome Sanger Institute, Cambridge, United Kingdom; Babraham Bioinformatics, Babraham Institute, CB22 3AT Babraham, United Kingdom; Epigenetics programme, Babraham Institute, CB22 3AT Babraham, United Kingdom; Artios Pharma, Babraham Research Campus, CB22 3AT Babraham, United Kingdom; Epigenetics programme, Babraham Institute, CB22 3AT Babraham, United Kingdom

## Abstract

TrAEL-seq is a robust method for profiling DNA replication genome-wide that works in unsynchronized cells and does not require drugs or nucleotide analogues. Here, we provide an updated method for TrAEL-seq that improves sample quality and includes multiplexing of up to six samples which dramatically improves throughput, and we validate TrAEL-seq in multiple mammalian cell lines. The updated protocol is straightforward and robust yet provides excellent resolution comparable to OK-seq in mammalian cell samples. High resolution replication profiles can be obtained across large panels of samples and in dynamic systems, for example during the progressive onset of oncogene induced senescence. In addition to mapping zones where replication initiates and terminates, TrAEL-seq is sensitive to replication fork speed, revealing effects of both transcription and proximity to replication Initiation Zones on fork progression. Although forks move more slowly through transcribed regions, this does not have a significant impact on the broader dynamics of replication fork progression, and instead replication forks accelerate across the first ∼1 Mb of travel irrespective of local transcriptional activity. We propose that this is a consequence of fewer replication forks being active later in S-phase when these distal regions replicate and there being less competition for replication factors.

## Graphical Abstract

**Graphical Abstract:**
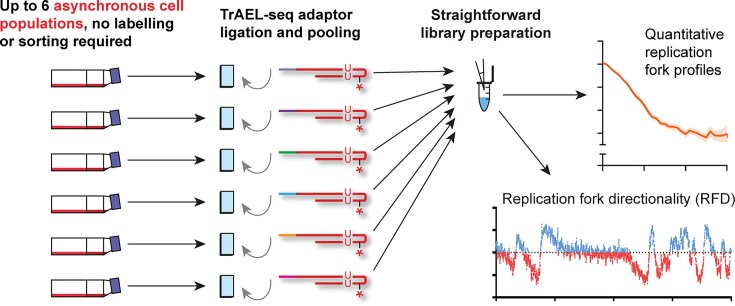

## Introduction

DNA replication is initiated by the firing of replication origins. Origins in budding yeast are exactly defined by specific DNA sequences but in mammalian cells are heterogeneously distributed and poorly defined with replication initiation events concentrated in, though not restricted to, large Initiation Zones of up to 150 kb [1–4]. The pattern of Initiation Zone usage varies between cell types, but is robust across multiple cells in a population, showing that Initiation Zone usage is specified even if individual initiation sites are not [1, 5, 6]. Discovering the set of Initiation Zones used in any given cell-type is a substantial undertaking, and we therefore have little understanding of how active Initiation Zones are specified in different cells, or how variable Initiation Zone usage really is.

Replication initiation can be profiled genome-wide by isolation of replication intermediates (OK-seq, SNS-seq, Bubble-seq) though these methods involve significant biochemical purifications [1, 2, 7]. Flow sorting of precise S-phase fractions identifies early origins though spatial resolution is lower (Repli-seq) [8, 9], while nucleotide analogue incorporation reveals initiation events if cells are synchronized or long read single molecule sequencing applied (INI-seq, DNAscent) [3, 4, 10]. DNA end mapping methods also yield replication profiles: GLOE-seq, which detects all single-stranded DNA ends can profile Okazaki fragments in a DNA ligase mutant [11], while TrAEL-seq, which detects free single stranded DNA 3′ ends in double stranded DNA has a strong selectivity for leading strand ends and provides DNA replication maps even in unsynchronized wildtype cells [12]. Finally, since DNA polymerase usage differs between leading and lagging strands, replication profiles can be constructed using polymerase mutants that incorporate excessive ribonucleotides on one strand or the other (PU-seq) [13, 14]. There is considerable discordance between these methods in the fine scale mapping of initiation events, but the general clustering events into Initiation Zones is widely reproduced [15]

Replication Initiation Zones are very widely spaced in some parts of mammalian genomes, and though late firing origins are present in these regions, DNA replication forks must still traverse large regions up to ∼1 Mb so particularly large replication zones are prone to incomplete replication resulting in fragile site expression if fork progression is impaired [1, 16, 17]. Replication fork speed is regulated to ensure complete, high fidelity replication, being increased in large replication zones where replication continues late in S-phase [18–20], but reduced under conditions of high oxidative damage or transcriptional load to minimize the hindrance to fork progression from obstacles [21, 22]. Although DNA fibre assays are widely used to measure replication fork processivity, these assays do not provide genomic information and therefore the effects of genome structure and localized obstacles cannot be determined. DNAscent, NanoForkSpeed, ForkML, and scEdU-seq offer genome-wide equivalents of these fibre assays, using nucleotide analogue incorporation to measure the distribution and speed of replication forks, though resolution of fork speed is limited by the analogue pulse time so the effect of individual obstacles is hard to determine [4, 10, 18, 23, 24]. TrAEL-seq has great potential in this regard as it can detect replication fork accumulation at pause sites with base-pair resolution in both asynchronous and synchronized populations [25, 26].

Impediments to DNA replication cause replication stress. Endogenous sources of replication stress include nucleotide depletion, unrepaired DNA lesions, mis-incorporated ribonucleotides, repetitive DNA sequences, secondary DNA structures, and collisions with transcription units (reviewed in [27]). The mutagenicity of transcription-replication conflicts has been particularly studied and arises both from direct interactions between the replication and transcription machinery, and from the replication inhibitory effects of R-loops [28–30]. Furthermore, exclusion of replication initiation from very large genes creates extended regions requiring contiguous replication; these are prone to under-replication and form fragile sites [16, 31, 32]. However, such mutagenic interactions are necessarily rare, indeed yeast and mammalian cells regulate transcription to minimize such transcription-replication conflicts so highly transcribed genes do not measurably delay replication forks in yeast [33–35]. Nonetheless, TrAEL-seq does detect transcription-replication interactions in yeast [12] and it remains to be clarified whether equivalent interactions occur in mammalian cells.

DNA replication is the target of both classic and targeted chemotherapeutics, and although we have an excellent understanding of the biochemical impact of these treatments, revealing how replication fork processivity is affected and how this is affected by features such as chromatin structure and transcription units remains extremely challenging. We previously demonstrated the utility of TrAEL-seq in profiling DNA replication in unsynchronized steady state cell populations, and here we present an updated version that improves sample quality and sample throughput while reducing cost. We validate TrAEL-seq in multiple human cell lines and show that TrAEL-seq reveals dynamic changes in replication fork processivity across the genome.

## Materials and methods

### Cell culture

Undifferentiated H9 human embryonic stem cells (hESCs) were maintained on Vitronectin-coated plates (Thermo Fisher Scientific, A14700) in TeSR-E8 media (StemCell Technologies, 05990) and Essential 8 Medium (Gibco, A1517001) under 5% O_2_, 5% CO_2_ at 37°C. DLD-1 cells were maintained in 75 cm^2^ flasks (Thermo Fisher Scientific, 156499 and 159910) in RPMI-1640 media (PAN Biotech, P04-18500) with 10% (v/v) fetal bovine serum (FBS) (Thermo Fisher Scientific, 10270-106), and 100 I.U/ml penicillin-streptomycin (Thermo Fisher Scientific, 15140122) under 5% CO_2_ at 37°C. Cells were passaged with trypsin–ethylenediaminetetraacetic acid (EDTA) 0.05% solution (Invitrogen, 25300062) and kept for a maximum of 20 passages. IMR90 ER:HRAS^G12V^ (IMR90 ER:RAS) fibroblasts were gifted from Prof. Masashi Narita at the CRUK Cambridge Institute. Cells were cultured in DMEM (Thermo Fisher Scientific, 31053028) with 10% (v/v) FBS (Thermo Fisher Scientific, 10270-106), 2 mM L-glutamine (Thermo Fisher Scientific, 25030081), 100 I.U/ml penicillin-streptomycin (Thermo Fisher Scientific, 15140122), and 1 mM sodium pyruvate (Thermo Fisher Scientific, 11360070) under 5% CO_2_ at 37°C. For passaging and collection, cells were detached with TrypLE™ Express Enzyme (Thermo Fisher Scientific, 12604013) and centrifuged at 300 × *g* for 3 min. For time course experiments, IMR90 ER:RAS cells were seeded in 10–15 cm dishes (Thermo Fisher Scientific, 150350) and the following day were treated with 4-hydroxy tamoxifen (4OHT; Sigma, H7904) at a final concentration of 100 nM. 4OHT was replenished every 2–3 days. Cells were fully senescent following 6 days of treatment with 4OHT. Hydroxyurea (Merck H78627) was used at 20–100 µM for 2 h, Ceralasertib (Merck TA9H11E41972) at 1 µM for 24 h, palbociclib (Thermo Fisher Scientific 16430568) at 16 nM for 24 h, triptolide (Thermo Fisher Scientific PG490) at 3 µM for 4 h.

### Mouse B-cell cultures

Spleens were isolated from C57Bl6 wild-type and Activation-Induced Demaminase (AID) knock-out mice obtained from the European Conditional Mouse Mutagenesis Program (*Aicda*^tm1a (EUCOMM)Hmgu^). All mice used were bred and maintained in the Babraham Institute Biological Support Unit (BSU) under pathogen-free conditions. Genotyping was performed using Transnetyx assays. Animal husbandry and experimentation complied with European Union and United Kingdom Home Office legislation and was approved by the Babraham Institute Animal Welfare and Ethical Review Body.

Splenic cell suspensions were prepared in RPMI 1640 media supplemented with 10% heat-inactivated FBS and 100U pen/strep using a 70 μm cell strainer and mechanical disruption. B-cells were enriched to >95% purity by magnetic depletion using a B-cell isolation kit as per manufactures instructions (Miltenyi Biotech). B cells were cultured at 0.2–0.4 × 10^6^ cells per ml in IMDM media supplemented with 10% heat-inactivated FBS, 100 U/ml pen/strep, and 50 μM β-mercaptoethanol, in the presence of LPS (25 μg/ml, *Escherichia coli* strain O55:B5, Sigma–Aldrich) and recombinant murine IL4 (20 ng/ml, Peprotech) for 3 days.

### EdU incorporation assay

Cells were treated with 10 µM EdU for 3 h before fixation with 2% paraformaldehyde for 15 min. After permeabilization for 15 min (Invitrogen, 00-8333-56), 1 ml of EdU stain (886 µl phosphate buffered saline, 4 µl 4 mM Sulfo-Cy5-Azide, 10 µl 200 mM CuSO_4_.5H_2_O, 100 µl 200 mg/ml ascorbic acid) was added to 600 µl cells and incubate 15 min at room temperature in the dark. Cells were washed with 2 ml of permeabilization reagent, resuspended in 1 ml of permeabilization reagent, passed through a 40 µM filter and stained with 1 µg/ml DAPI.

### TrAEL-seq adaptor preparation

Multiplexing variants of TrAEL-seq adaptor 1 were synthesized and PAGE purified by Integrated DNA Technologies (IDT). Sequences for TrAEL-seq adaptors are:

TrAEL-seq adaptor 1 multiplexing index 1: /5Phos/GACTNNNNNNNNAGATCGGAAGAGCGTCGT GTAGGGAAAGAGTGUAGC A/iBiodT/TGCUACACTCTTT CCCTACACGACGCTCTTCCG

TrAEL-seq adaptor 1 multiplexing index 3: /5Phos/CAAGNNNNNNNNAGATCGGAAGAGCGTCGT GTAGGGAAAGAGTGUAGC A/iBiodT/TGCUACACTCTTT CCCTACACGACGCTCTTCCG

TrAEL-seq adaptor 1 multiplexing index 5: /5Phos/CCTTNNNNNNNNAGATCGGAAGAGCGTCGT GTAGGGAAAGAGTGUAGC A/iBiodT/TGCUACACTCTTT CCCTACACGACGCTCTTCCG

TrAEL-seq adaptor 1 multiplexing index 6: /5Phos/GGAANNNNNNNNAGATCGGAAGAGCGTCGT GTAGGGAAAGAGTGUAGC A/iBiodT/TGCUACACTCTTT CCCTACACGACGCTCTTCCG

TrAEL-seq adaptor 1 multiplexing index 7: /5Phos/GCACNNNNNNNNAGATCGGAAGAGCGTCGT GTAGGGAAAGAGTGUAGC A/iBiodT/TGCUACACTCTTT CCCTACACGACGCTCTTCCG

TrAEL-seq adaptor 1 multiplexing index 8: /5Phos/TGGCNNNNNNNNAGATCGGAAGAGCGTCGT GTAGGGAAAGAGTGUAGC A/iBiodT/TGCUACACTCTT TCCCTACACGACGCTCTTCCG

where /5Phos/ indicates 5′ phosphate and /iBiodT/ indicates Biotin-dT. Adaptors were adenylated using the 5′ DNA adenylation kit (NEB, E2610S) as follows: 500 pMol DNA oligonucleotide, 5 µl 10 × 5′ DNA Adenylation Reaction Buffer, 5 µl 1 mM ATP, 5 µl Mth RNA Ligase in a total reaction volume of 50 µl were incubated for 1 h at 65°C then 5 min at 85°C. The reaction was extracted with phenol:chloroform pH 8, then ethanol precipitated with 10 µl 3M NaOAc, 1 µl GlycoBlue (Thermo AM9515), 330 µl 100% (v/v) ethanol, and resuspended in 50 µl 0.1× TE (1xTE is 10mM Tris pH8, 1mM EDTA). All adenylated adaptors were stored at −30°C.

TrAEL-seq adaptor 2 was purchased PAGE purified from Merck or (latterly) IDT. Sequence:

/5Phos/GATCGGAAGAGCACACGTCTGAACTCCAGT CUUUUGACTGGAGTTCAGAC GTGTGCTCTTCCGAT C*T

where /5Phos/ indicates 5′ phosphate and * indicates a phosphorothioate bond. Adaptor was annealed in a total reaction volume of 200 µl with 20 µl 100 pM/µl oligonucleotide and 20 µl 10× T4 DNA ligase buffer (NEB, B0202S) and heated at 95°C for 5 min. The reaction was removed from heat and left to cool to room temperature over approximately 2 hr.

### Embedding cells in agarose

Cells were resuspended in a volume of 60 µl L-buffer (100 mM EDTA, pH 8, 10 mM Tris, pH 7.5, 20 mM NaCl) per plug (∼1 × 10^6^ cells per plug) before distribution into 2 ml round-bottom tubes (one tube per plug). Each tube was incubated at 50°C for 5 min, meanwhile an aliquot of CleanCut Agarose (Bio-Rad, 1703594) was heated until molten. To the 60 µl cell suspension, 40 µl of molten Cleancut Agarose was added and immediately vortexed for 10 s, then 100 µl of solution was transferred into a disposable plug mould (Bio-Rad, 170-3713). The plug mould was placed on its side at 4°C for 30 min before pushing plugs out into 2 ml tubes containing 500 µl of fresh mammalian digestion buffer [100 mM EDTA, pH 8, 10 mM Tris, pH 7.5, 20 mM NaCl, 1% (v/v) N-lauroyl sarcosine, 0.1 mg/ml proteinase K]. Plugs were incubated at 50°C overnight. Plugs were then rinsed with 1 ml TE, then washed three times with 1 ml TE for 1–2 hr at room temperature with rocking, and 10 µl of 100 mM PMSF (Merck, 93482) was added to the second and third washes from 100 mM stock. Plugs were incubated in 200 µl 1× TE containing 1 µl of RNase T1 (Thermo, EN0541) for 1 h at 37°C. The RNase solution was then removed and plugs were washed with 1 ml 1× TE for 1 h before storing at 4°C in 1 ml 1× TE until use (plugs are stable for > 1 year). Yeast cells were embedded and processed as described [12].

### A-tailing and adaptor 1 ligation

Half of an agarose plug was used per library by cutting with a razor blade. From this point onwards the use of the term plug relates to use of a half plug. Plugs were placed in 2 ml round bottomed tubes, as with all reactions prior to agarose digestion to prevent the plug from breaking, and equilibrated once in 100 µl 1 × TdT buffer (NEB, B0135S) for 30 min at room temperature, then incubated for 2 hr at 37°C in 100 µl 1 × TdT buffer containing 0.4 µl 100 mM ATP (Roche, 11140965001) and 2 µl Terminal Transferase (NEB, M0315L). Plugs were rinsed with 1 ml Tris buffer (10 mM Tris–HCl, pH 8.0), then equilibrated in 100 µl 1 × T4 RNA ligase buffer (NEB, B0216S) containing 40 µl 50% (w/v) PEG 8000 for 1 h at room temperature then incubated overnight at 25°C in 100 µl 1 × T4 RNA ligase buffer (NEB, B0216S) containing 40 µl 50% (w/v) PEG 8000, 1 µl 10 pM/µl TrAEL-seq adaptor 1 with multiplex index and 1 µl T4 RNA ligase 2 truncated KQ (NEB M0373L). Plugs were then rinsed with 1 ml Tris buffer, transferred to individual 15 ml tubes and washed three times in 10 ml Tris buffer with rocking at room temperature for 1–2 h each, then washed again overnight under the same conditions.

### Agarose digestion and DNA extension

Up to 3 plugs with different multiplex indexes were transferred to 1.5 ml Eppendorf tubes and equilibrated for 15 min with 1 ml agarase buffer (10 mM Bis–Tris–HCl, 1 mM EDTA, pH 6.5) before the supernatant was removed and replaced with 50 µl agarase buffer per plug. Plugs were melted for 20 min at 65°C, then transferred for 5 min to a heating block pre-heated to 42°C, followed by addition of 1 µl per plug β-agarase (NEB, M0392S) and flicking to mix the contents without allowing the sample to cool. Tubes were then incubated at 42°C for 1 h. DNA was ethanol precipitated by adding 25 µl per plug 10 M NH_4_OAc, 1 µl per plug GlycoBlue, and 330 µl per plug 100% (v/v) ethanol and vortexing, after which the pellet was washed in 70% (v/v) ethanol and then resuspended in 10 µl per plug 0.1× TE (mammalian DNA samples were heated at 65°C for 10 min to aid resuspension). DNA from up to six plugs with different multiplex indexes were combined in a single tube at this point. Next 40 µl per plug of reaction mix containing 5 µl isothermal amplification buffer (NEB, B0537S), 3 µl 100 mM MgSO4 (NEB, B1003S), 2 µl 10 mM dNTPs, and 1 µl Bst 2 WarmStart DNA polymerase (NEB, M0538S) was added to the sample and incubated 30 min at 65°C before ethanol precipitation with 12.5 µl per plug 10 M NH_4_OAc, 1 µl per plug GlycoBlue, and 160 µl per plug 100% (v/v) ethanol and re-dissolving the pellet in 130 µl total volume (irrespective of the number of plugs) 1× TE for 10 min at 65°C.

### DNA shearing and streptavidin bead binding

From this point onwards the protocol is identical for 1–6 multiplexed plugs.

The DNA was transferred to an AFA microTUBE (Covaris, 520045) and fragmented in a Covaris E220 using duty factor 10, PIP 175, Cycles 200, Temp 11°C, then transferred to a 1.5 ml tube containing 8 µl pre-washed Dynabeads MyOne streptavidin C1 beads (Thermo Fisher Scientific, 65001) re-suspended in 300 µl 2×TN (10 mM Tris, pH 8, 2 M NaCl) along with 170 µl water (total volume 600 µl) and incubated 30 min at room temperature on a rotating wheel. Beads were washed once with 500 µl 5 mM Tris, pH 8, 0.5 mM EDTA, 1 M NaCl, 5 min on wheel and once with 500 µl 0.1× TE for 5 min on wheel before re-suspension in 25 µl 0.1× TE.

### Sequencing library preparation

TrAEL-seq adaptor 2 was added using a modified NEBNext Ultra II DNA kit (NEB, E7645S): 3.5 µl NEBNext Ultra II End Prep buffer, 1 µl 1 ng/µl sonicated salmon sperm DNA (this is used as a carrier) and 1.5 µl NEBNext Ultra II End Prep enzyme were added and reaction incubated 30 min at room temperature and 30 min at 65°C. After cooling for 15 min, 1.25 µl 10 pM/µl TrAEL-seq adaptor 2, 0.5 µl NEBNext ligation enhancer and 15 µl NEBNext Ultra II ligation mix were added and incubated for 30 min at room temperature. The reaction mix was removed and beads were rinsed with 500 µl wash buffer (5 mM Tris, pH 8, 0.5 mM EDTA, 1 M NaCl), then washed twice with 1 ml wash buffer for 10 min on rotating wheel at room temperature and once for 10 min with 1 ml 0.1× TE. Libraries were eluted from beads with 11 µl 1× TE and 1.5 µl USER enzyme (NEB, M5505S) for 15 min at 37°C, then again with 10.5 µl 1× TE and 1.5 µl USER enzyme (NEB, M5505S) for 15 min at 37°C, and the two eluates were combined. An initial test amplification was used to determine the optimal cycle number for each library. For this, 1.25 µl library was amplified in 10 µl total volume with 0.4 µl each of the NEBNext Universal and any NEBNext Index primer with 5 µl NEBNext Ultra II Q5 polymerase chain reaction (PCR) master mix. Cycling program: 98°C for 30 s, then 14–16 cycles (16 for 1–3 multiplexed libraries, 15 for 4–5, 14 for 6) of (98°C for 10 s, 65°C for 75 s), 65°C for 5 min. Test PCR was cleaned with 8 µl AMPure XP beads (Beckman, A63881) and eluted with 2.5 µl 0.1× TE. One microliter of test PCR was examined on a Bioanalyser high sensitivity DNA chip (Agilent 5067-4626). The ideal cycle number was selected to bring the final library to final concentration of 2–4 nM, noting that the final library would be 2–3 cycles more concentrated than the test sample. 21 µl of library was then amplified with 2 µl each of NEBNext Universal and the chosen Index primer and 25 µl NEBNext Ultra II Q5 PCR master mix using the same conditions as described above for calculated cycle number. The amplified library was cleaned with 40 µl AMPure XP beads (Beckman A63881) and eluted with 26 µl 0.1× TE, then 25 µl of eluate was purified with 20 µl AMPure XP beads and eluted with 11 µl 0.1× TE. Final libraries were quality controlled and quantified by Bioanalyser (Agilent, 5067-4626) and KAPA qPCR (Roche, KK4835). Libraries were sequenced on an Illumina NextSeq 500 or (latterly) Element Biosciences AVITI as Single End by the Babraham Institute Genomics facility (35–40 million reads per mammalian sample).

A detailed TrAEL-seq protocol is provided in Supplemental File 1 .

### Data processing and mapping

Processing and mapping of all raw sequence files was carried out by the Babraham Bioinformatics facility using the updated pipeline developed by Laura Biggins (https://github.com/laurabiggins/TrAEL-seq). This pipeline includes trimming of the poly (T) tail, separation of multiplexed sets, separation into T and no-T datasets, copy number aware UMI deduplication and mapping. For deduplication, high quality mapped reads (always unique) were deduplicated by mapped position and UMI, whereas for reads with mapping quality ≤ 20 only the UMI was used for deduplication but since an 8 bp UMI only has 65k combinations (smaller than the library) the first 10 bases of the unique read sequence are added to the UMI before deduplication. Human and mouse libraries were mapped to GRCh38 and GRCm38, respectively. All *Saccharomyces cerevisiae* yeast libraries were mapped to the R64-1-1 genome with the 2-micron plasmid assembly.

### Data processing

Mapped BAM files were imported into Seqmonk (v1.48.2) and reads were trimmed down to 1 nucleotide at the 5′ end, representing the last nucleotide 5′ of the strand break. Quality filtering was set ≥20 for most analysis but this cutoff was removed for analysis of multi-copy regions. For read count comparisons within multiplexed sets, no normalization was applied as the read count distribution within a multiplexed set should reflect the relative distribution of TrAEL-seq reads across the multiplexed samples. To compare across multiplexed sets, a total read count normalization was applied to whole multiplexed sets, maintaining the relative distribution of read counts for samples within each multiplexed set.

RFD plots were calculated using the new SeqMonk strand bias quantitation option introduced for this purpose. Note that this option generates data from −100 to +100 which was scaled to −1 to +1 for plots for consistency with published RFD plots. Data was analysed in 20 kb windows spaced every 2 kb, split into positive and negative values and plotted on the same graph in different colours using GraphPadPrism.

Locations of Initiation Zones were determined using OKseqHMM [36]. TrAEL-seq reads were then summed in 50 kb windows spaced every 50 kb for the relevant datasets, filtering regions with aberrant counts (e.g. line-specific CNVs and peri-centromeres), and exported as Annotated Probe Reports. These were processed using the ‘read_distance_distribution2.R’ script (https://github.com/laurabiggins/TrAEL-seq) which calculates mean read count as a function of distance from the nearest of a set of defined features (in this case the centres of Initiation Zones), as well as the 96% confidence interval and the number of regions used for the calculation. Datasets were truncated at 3 Mb as very few regions of mammalian genomes are >3 Mb from an Initiation Zone and therefore the confidence interval for these regions is very large.

## Results and discussion

### Multiplexed TrAEL-seq in mammalian cells

TrAEL-seq uses a simple Terminal Transferase-mediated ligation to capture and sequence free single stranded 3′ DNA ends. It was designed to map DNA breaks, but has remarkably high affinity for the leading strand of DNA replication forks, providing profiles of replication fork directionality (RFD) across the genome in untreated asynchronous populations of wild-type cells [12]. We attribute this unanticipated selectivity for replication forks to the conformational flexibility of fork structures after proteins are removed, which allows a thermodynamic equilibrium of the classical fork structure with a reversed form that exposes the 3′ end of the leading strand (Supplementary Fig. S1A).

**Figure 1.**
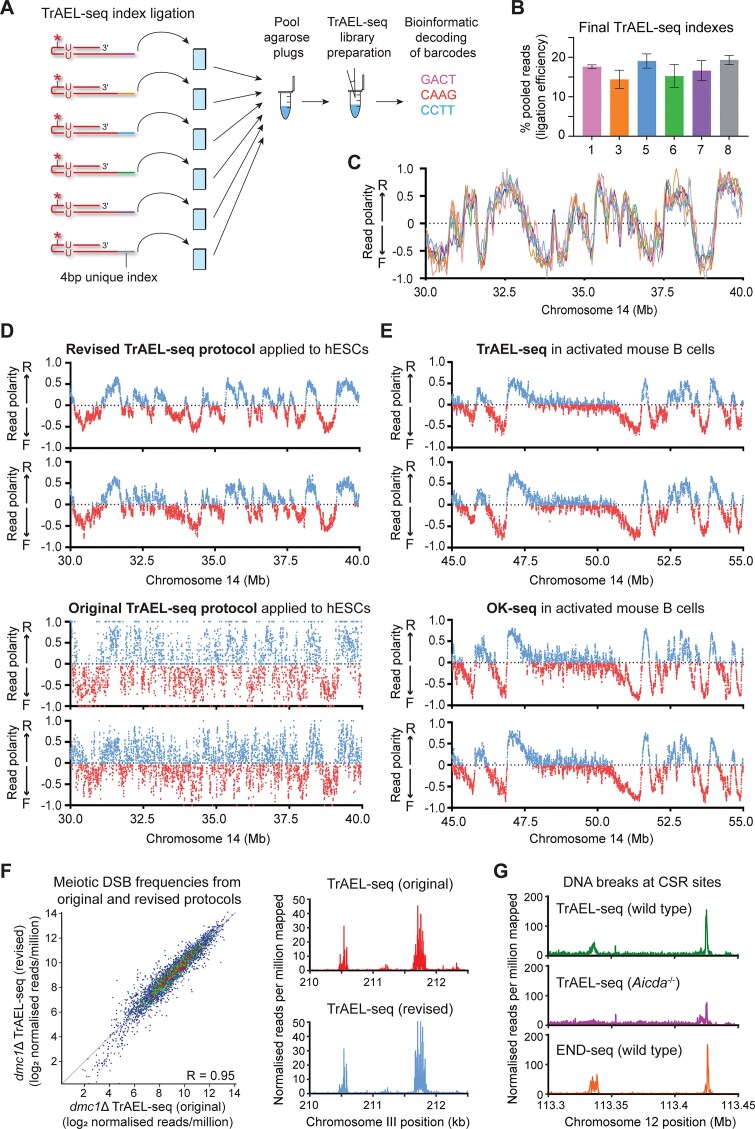
Multiplexed TrAEL-seq yields high quality replication profiles in mammalian cells. (**A**) Schematic of multiplexed TrAEL-seq—barcoded adaptors are incorporated early in protocol allowing samples to be pooled for processing. Multiple pools can be generated over a period of weeks then all processed into libraries together. (**B**) Read counts from TrAEL-seq adaptors with six different indexes ligated competitively to one DNA sample of DLD-1 cells in a single reaction. Some barcodes affect ligation efficiency as the barcode is placed at the ligation junction, but these barcodes show equivalent ligation efficiencies. Error bars ± 1 SD; *n* = 4. (**C**) Replication fork directionality (RFD) plots of an example chromosomal region obtained from the competitive ligation experiment in panel (B). RFD is calculated from read polarity as (R − F)/(R + F), such that positive values represent forks moving left→right and negative values represent forks moving right→left. RFD values were calculated in 20 kb windows. (**D**) RFD plots of TrAEL-seq data from hESCs showing two biological replicates generated under the revised multiplexed protocol compared to two biological replicates from our original TrAEL-seq study [12]. RFD values calculated as in panel (C) in 20 kb windows spaced every 2 kb. (**E**) RFD plots comparing TrAEL-seq data generated using the new protocol to published OK-seq data for activated mouse B cells [37, 38], performed as in panel (D). (**F**) Comparison of meiotic DSB profiles from *dmc1*Δ cells profiled using the original TrAL-seq protocol [12] and equivalent cells profiled using the revied protocol [39]. Scatter plot shows log transformed normalized read counts at 3907 Spo11 cleavage hotspots annotated by Mohibullah and Keeney [40], while panels on the right show an example 2.5 kb region in which reads were quantified at base-pair resolution. (**G**) TrAEL-seq profiles of LPS-activated splenic B cells from wild-type and *Aicda*^−/−^ mice, reads are summed in 100 bp windows. END-seq data for equivalent wild-type cells from Canela *et al.* [41] is shown for comparison.

The throughput of TrAEL-seq library synthesis can be increased by adding a 4 nucleotide in-line barcode to the first TrAEL-seq adaptor allowing libraries to be pooled for processing after initial adaptor ligation (Fig. 1A). Although we previously multiplexed pairs of samples in this way [12], the resulting barcode efficiencies were poor so we redesigned the adaptors and changed manufacturer, resulting in dramatically better barcode fidelity and minimal unassigned reads. Of nine barcodes tested, six showed equivalent, high TrAEL-seq efficiency, providing similar read numbers when ligated competitively to a single sample of DLD-1 cells (Fig. 1B and Supplementary Fig. 1B), and RFD profiles obtained through this six-way multiplexing procedure were indistinguishable (Fig. 1C).

We rebuilt the TrAEL-seq bioinformatic pipeline to separate reads by inline barcode prior to mapping and introduced a new copy number-aware UMI deduplication step. We further added a quality control routine to ensure TrAEL-seq reads stem from DNA as in theory TrAEL-seq can also detect RNA; although we have observed no evidence of read contamination from abundant RNA species, TrAEL-seq may detect RNA that is complexed with DNA. TrAEL involves tailing of DNA 3′ ends with ATP following by adaptor ligation using an RNA ligase, and could therefore detect any nucleic acids terminating with a 3′ RNA nucleotide through terminal transferase-independent ligation of adaptor to such ends. DNA-derived reads have an invariant T at the first nucleotide from the A-tailing, whereas RNA-derived reads could start with any nucleotide, so by splitting reads into those which start with a T and those which do not, unexpected sites of read accumulation can be assigned to DNA or RNA depending on representation in the no-T fraction. No-T reads constitute up to 5% of the mapped reads in some libraries, and although these largely follow the replication pattern, it is possible that other features such as nascent transcripts and R-loops contribute at some sites.

TrAEL-seq libraries generated using the multiplexed protocol are generally of much higher quality and yield more unique reads than libraries made using the original TrAEL-seq protocol, allowing high resolution analysis (Fig. 1D). The improvement in quality was unexpected but is consistent and likely stems from the refined adaptor sequences as opposed to the multiplexing itself. Reproducibility and specificity are excellent as RFD plots generated from unsynchronized mammalian cell samples show very similar patterns of RFD and Initiation Zone usage between biological replicates, but clear differences in Initiation Zone usage between lines as expected (Supplementary Fig. S1C). Replication profiles derived by TrAEL-seq also closely match those generated by OK-seq, as evident in a comparison of published TrAEL-seq and OK-seq data generated from activated mouse B cells in different laboratories (Fig. 1E). Note that the directionality of replication forks detected by TrAEL-seq and OK-seq is the same as TrAEL-seq labels the 3′ end of the leading strand whereas OK-seq labels the 5′ end of Okazaki fragments [37, 38].

TrAEL-seq uses a Terminal Transferase-assisted ligation to capture and sequence single-stranded 3′ DNA ends, and was designed to detect DNA double strand break ends such as the meiotic Spo11 cleavages in *dmc1*Δ *S. cerevisiae* cells [12]. We therefore compared the original *dmc1*Δ dataset to a recently published dataset generated using the revised protocol (Fig. 1F) [12, 39], and observed excellent concordance both of site usage and meiotic break pattern. To test DSB detection in mammalian cells, we then examined class switching in LPS-activated mouse B cells. TrAEL-seq detected known AID-induced breaks at the µ and γ1 switch, which were profoundly reduced in cells from *Aicda*^−/−^ mice lacking AID (Fig. 1G). Curiously, the signal was not entirely abrogated in the absence of AID, and we attribute the residual peak at the γ1 site to replication fork stalling at R-loops that form at class switch sites. We also note that END-seq provides superior signal to noise for DSBs in this application albeit with lower throughput [41], because TrAEL-seq also detects replication forks at and around the class switch regions in these highly proliferative cells. Overall, the revised multiplexed TrAEL-seq protocol increases the quality, throughput and specificity of TrAEL-seq while maintaining the capability of the original method to assay complex systems without synchronization, sorting, genetic modification, or labelling. To aid in setting up the method, we provide a detailed protocol including solutions to common TrAEL-seq problems (Supplemental File 1). Furthermore, ‘How to’ videos for making and handling agarose plugs, as well as the latest protocol version are available at https://www.babraham.ac.uk/our-research/epigenetics/jon-houseley/protocols.

### Replication fork redistribution during oncogene induced senescence

**Figure 2.**
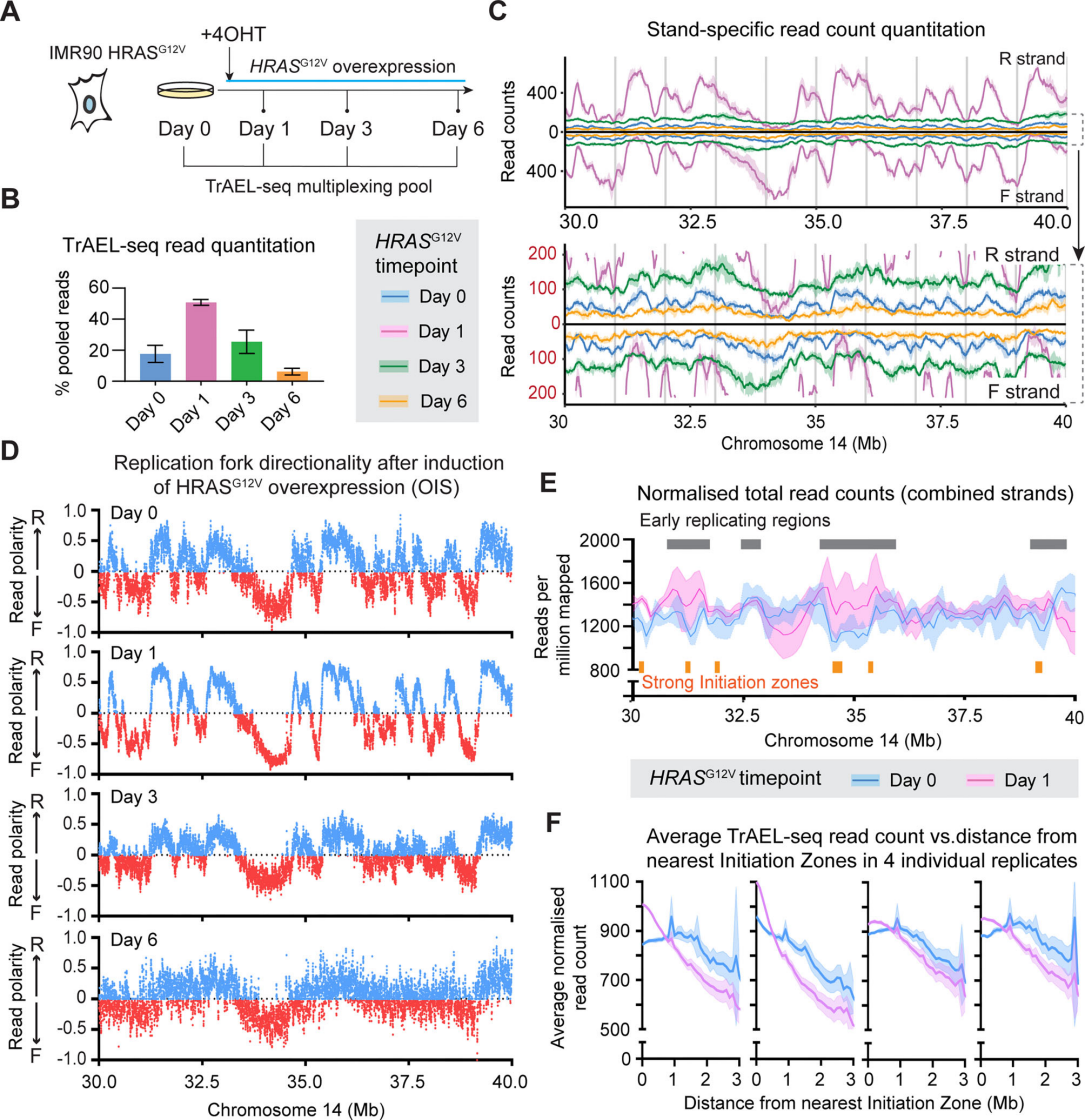
Multiplexed TrAEL-seq allows replication profiling in complex systems. (**A**) Schematic of OIS timecourse in IMR90 with tetracyclin inducible HRAS^G12V^. (**B**) Number of reads obtained from TrAEL-seq libraries at each time point. Each replicate time course was multiplexed in a separate TrAEL-seq library, with indexes rotated to avoid index-specific effects. Error bars ± 1 SD; *n* = 4. (**C**) Quantitative profiles of TrAEL-seq reads over an example chromosomal region at different timepoints leading to OIS. Reverse and Forward reads (which capture replication forks moving left→right and right→left, respectively) are shown, separately. Solid line shows mean signal, shaded band ± 1 SD; *n* = 4. (**D**) RFD plots across the same example chromosome region shown in panel (C), separated by OIS timepoint. Calculated in 10 kb windows spaced every 1 kb. (**E**) Total TrAEL-seq read count across an example chromosomal region for day 0 and day 1 of OIS timecourse. Solid line shows mean signal, shaded band ± 1 SD; *n* = 4. (**F**) Plots of total TrAEL-seq at increasing distance from replication Initiation Zones. Each plot shows a single biological replicate, solid line shows mean value, shaded band 95% confidence interval.

We applied multiplexed TrAEL-seq to profile the dynamic transitions in DNA replication during the onset of oncogene induced senescence (OIS). Expression of HRAS^G12V^ in human IMR90 fibroblasts initially causes hyper-replication (day 1), but this is unsustainable and proliferation soon declines (day 3), entirely ceasing by day 6 as cells enter senescence (Fig. 2A) [42, 43]. We acquired four replicate OIS time courses (time points at days 0, 1, 3, and 6), with individual libraries in each multiplexed replicate set showing expected differences in read count given the varying proliferation state of the cells over time (Fig. 2B): TrAEL-seq reads increased dramatically from day 0 to day 1 but were very rare by day 6 (Fig. 2C). Pooling the replicate sets yielded high resolution RFD plots particularly at day 1, with progressively decreasing resolution to day 6 as expected since very few cells are replicating by this time (Fig. 2D), but to our surprise we did not observe any change in Initiation Zone usage between day 0 and day 1 despite the dramatic increase in replication (Fig. 2C and D, and Supplementary Fig. S2A).

However, read count distribution across chromosomes was altered as hyper replication commenced, with peaks appearing at many early replicating genomic regions (Fig. 2E). Genome wide comparison with replication timing data for HCT116 cells [44] reveals that although average read count across the genome does not change markedly, the read count in early replicating regions is much higher on day 1 than on day 0 while read count in late replicating regions is much lower (Supplementary Fig. S2B). The strongest Initiation Zones are found in early replicating regions, and peaks of TrAEL-seq reads were often coincident with strong Initiation Zones at day 1. To quantify this effect, we calculated the average read count in genomic regions as a function of distance from the nearest Initiation Zone, and observed in all four replicate sets that the normalized read count on day 1 was greater within ∼0.6 Mb of Initiation Zones (*p *= 0.005 by *t*-test of Area Under Curves (AUCs)) but relatively depleted at regions > 1 Mb from Initiation Zones (*p *= 0.049 by *t*-test of AUCs), which are late replicating (Fig. 2F). The strength of this effect was variable between biological replicates, but this is to be expected considering the dynamic changes in proliferation both before and after day 1.

Multiplexed TrAEL-seq can therefore profile DNA replication in complex, dynamic systems to reveal both Initiation Zone usage and replication fork distribution. TrAEL-seq read density varies with replication state and is almost absent in non-proliferating day 6 cells (Fig. 2C and D), and furthermore TrAEL-seq read orientation is highly polarized around Initiation Zones at both day 0 and day 1 (Fig. 2C). Therefore, the vast majority of TrAEL-seq reads most likely arise from replication forks rather than noise or some other biological process, and although high levels of fork cleavage soon after initiation could give rise to an accumulation of polarized reads around Initiation Zones this is unlikely given the high proliferation rate of the IMR90 cells at day 1. We therefore attribute this accumulation of TrAEL-seq reads around Initiation Zones to an uneven distribution of replication forks across the genome.

### Variation in replication fork processivity over the human genome

**Figure 3.**
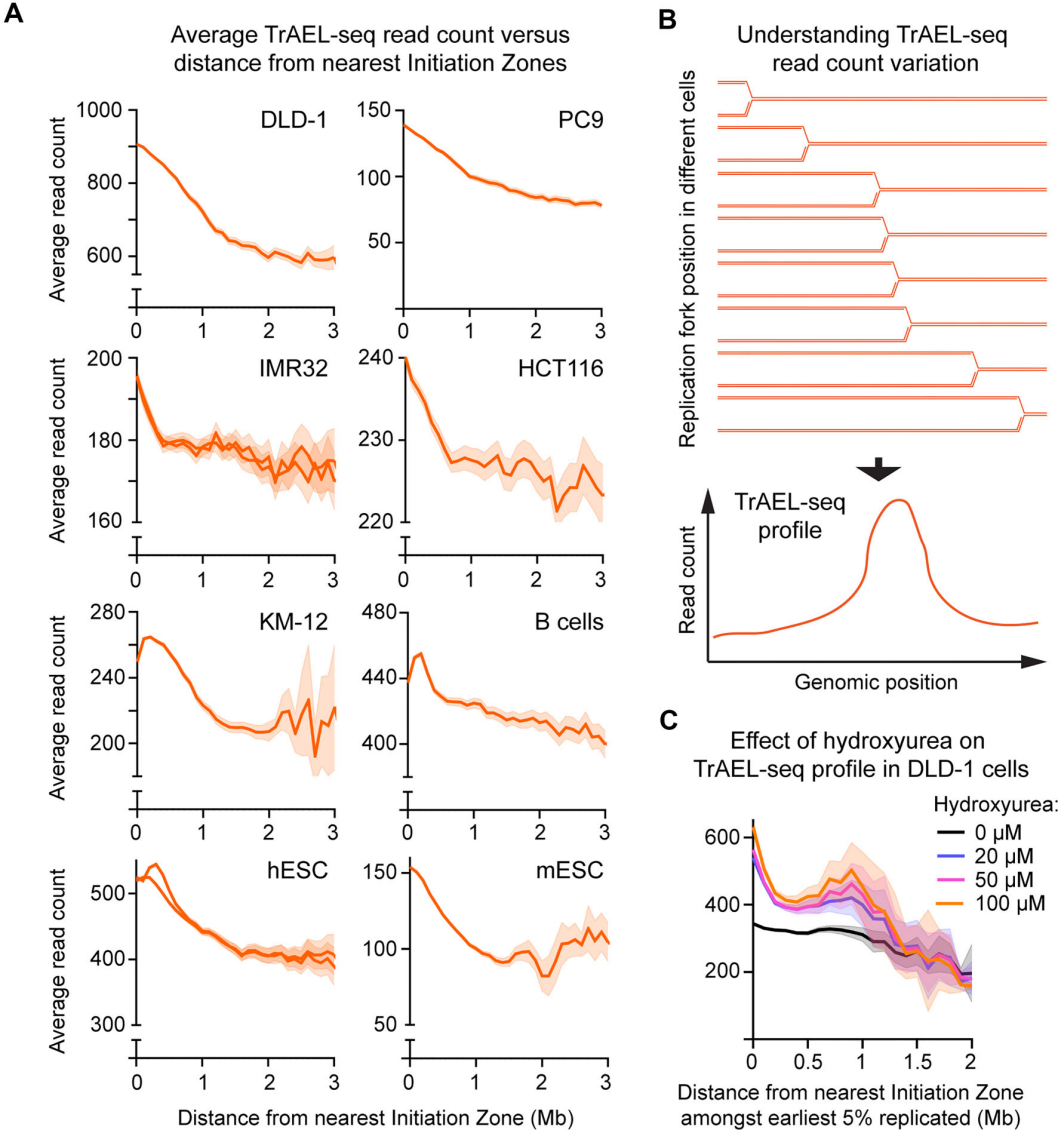
TrAEL-seq profiling of variable replication fork density. (**A**) Plots of total TrAEL-seq read count at increasing distance from replication Initiation Zones. Each plot shows a different cell line, multiple lines indicate multiple biological replicates where available, solid line shows mean value, shaded band 95% confidence interval. B cell sample shows splenic B cells from C57BL/6 mice activated in culture. Read counts were summed in 50kb windows spaced every 50 kb, with regions of altered copy number or aberrant read count removed. Distance was calculated from Initiation Zones determined using OKSeqHMM [36], and average read count determined in 100 kb windows of distance from the centre of initation zones. Published datasets are from [25, 38, 45, 46], note that IMR32 data was generated prior to the implementation of the revised TrAEL-seq protocol, and is therefore derived from lower resolution datasets. (**B**) Schematic showing how TrAEL-seq read density variations arise from variations in replication fork speed averaged across a population. (**C**) Plot of total TrAEL-seq read count at increasing distance from replication Initiation Zones for DLD-1 cells treated with 0, 20, 50, and 100 µM hydroxyurea for 2 h. TrAEL-seq read accumulation occurs exclusively at early firing Initiation Zones so only Initiation Zones in the earliest replicating 5% of the genome are included (based on HCT116 Repli-Seq [44]), and genomic regions that are closer to a later-firing initiation zone than to an initiation zone in the earliest 5% have been filtered out. Note that only 2 Mb from these initiation zones is considered as almost no genomic regions are >2 Mb from one of the earliest firing 5% initiation zones without being closer to a later firing initiation zone.

As the change in TrAEL-seq read distribution occurred in response to an oncogenic stimulus, we examined datasets for other transformed lines and observed a read accumulation around Initiation Zones to a greater or lesser extent for all tested including DLD-1, IMR32, PC9, HCT116, and KM-12 (Fig. 3A). However, this phenomenon is not unique to transformed cells or in fact to human cells as we also observed increased TrAEL-seq read density near Initiation Zones in hESCs, in mouse embryonic stem cells and in activated mouse splenic B cells (Fig. 3A).

Importantly, all of these samples derive from unsynchronized, unsorted cell populations assayed at steady state proliferation. Furthermore, EdU incorporation in DLD-1 cells does not indicate an enrichment in early S-phase (Supplementary Fig. S3A). Every part of the genome must replicate once per cell cycle, so in populations of unsynchronized cells the density of replication forks should be uniform across the genome as long as replisomes travel at a uniform speed. The fact that more replication forks are detected in some regions of the human genome indicates that replication forks spend longer replicating these regions than other regions of equivalent size. In other words, higher TrAEL-seq read density indicates a genomic region in which replication forks are either pausing more frequently or moving more slowly (Fig. 3B). To test this, we treated DLD-1 cells with increasing doses of hydroxyurea as a fork slowing/stalling agent (Fig. 3C), and observed that TrAEL-seq read density around early firing replication Initiation Zones (earliest 5% in the genome based on HCT116 data [44]) increased in a dose dependent manner. Increases in read density were restricted to the earliest-firing initiation zones (Supplementary Fig. S3B), consistent with the use of hydroxyurea as an early S-phase synchronization agent [47–50], which suggests that replication forks are most susceptible to hydroxyurea in early S-phase. Read count at later firing initiation zones (after the first 20%) is depressed by higher concentrations of hydroxyurea as fork stalling at earlier initiation zones will trigger the S-phase checkpoint (Supplementary Fig. S3B) (reviewed in [51]).

To ensure using orthogonal methods that higher read counts around Initiation Zones are not a TrAEL-seq artefact, we first performed SSB-seq on DLD-1 cells, which should detect replication forks by extending and labelling both leading and lagging strand ends [52]: in accord with the TrAEL-seq data, SSB-seq reads are also biased towards Initiation Zones (Supplementary Fig. S3C). We also found that OK-seq reads in published B cell data are biased towards Initiation Zones (Supplementary Fig. S3D) [37]; other explanations of this are possible as OK-seq read count could be affected by ligation rate, but it is reassuring that OK-seq reads show the same genomic distribution as TrAEL-seq reads. An increase in TrAEL-seq signal could also be explained by more frequent or more stable replication fork reversal events; however, we saw little change in the read distribution around Initiation Zones in SW-48 cells lacking the fork reversal factor SMARCAL1 (Supplementary Fig. S3E), so we consider it unlikely that this is a major contributor. Most importantly, however, the acceleration of replication forks across S-phase has been independently observed using single molecule and single cell approaches [18, 20, 24].

These findings combined with our previous work show that TrAEL-seq read density depends on local replication fork processivity [12, 25, 26], and indicate that the decreasing TrAEL-seq density with increasing distance from Initiation Zones we observe in mammalian cell lines results from replication forks moving faster in regions further from Initiation Zones that are replicated late in S-phase.

### Replication-transcription collisions do not explain varying replisome speed

**Figure 4.**
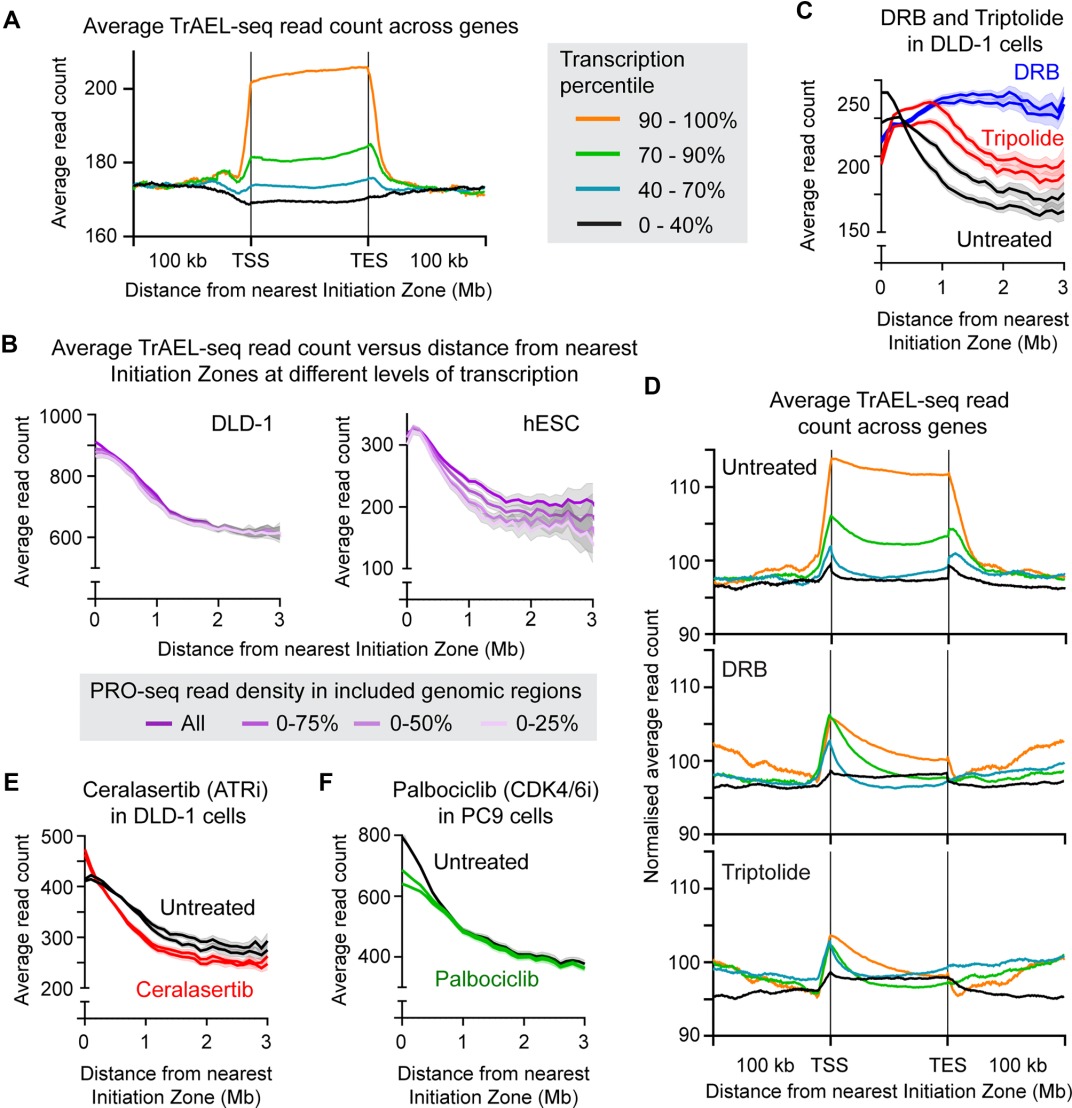
Causes of replication fork processivity differences across the genome. (**A**) Metaplot of TrAEL-seq read count in DLD-1 cells averaged across genes ± 100 kb. Genes are stratified for transcriptional activity based on PRO-seq into 0%–40%, 40–70%, 70%–90%, and 90%–100% categories. Profiles were normalized individually to make background read counts as close as possible. (**B**) Plots of total TrAEL-seq read count at increasing distance from replication Initiation Zones, stratified for nascent transcription level by PRO-seq. Analysis was performed as in Fig. 3A, but the genomic windows included were filtered to remove the top 25%, 50%, or 75% of regions based on PRO-seq read count. (**C**) Total TrAEL-seq read count at increasing distance from replication Initiation Zones in untreated DLD-1 cells or cells treated for 2 h with 100 µM DRB or for 4 h with 3 µM triptolide. (**D**) Metaplot of TrAEL-seq read count over genes ± 100 kb as in panel (A) in DLD-1 cells ± DRB and triptolide (datasets as in panel (C), data is an average of the two biological replicates shown). (**E**) Total TrAEL-seq read count at increasing distance from replication Initiation Zones in untreated DLD-1 cells and cells treated for with 1 µM Cerelasertib for 24 h. (**F**) Total TrAEL-seq read count at increasing distance from replication Initiation Zones in untreated PC9 cells and cells treated for 24 h with 16 nM palbociclib.

Many studies have suggested that interactions with the transcription machinery can impair fork processivity and, based on published DLD-1 PRO-seq data, we find that TrAEL-seq reads are enriched ∼20% in the top 10% of highly expressed genes compared to surrounding sequence indicating that replication forks move more slowly through highly transcribed genes (Fig. 4A) [53]. Reads lacking an initial T, which are predicted to be RNA-derived are slightly more enriched presumably through detection of nascent transcripts, but the contribution of these to the overall enrichment is <10% (Supplementary Fig. S4A). Furthermore, an equivalent signal in the T reads was observed when considering reads orientated forward or reverse to the direction of transcription, of which only the reverse reads could conceivably arise from detection of the nascent RNA (Supplementary Fig. S4B). Enrichment of TrAEL-seq reads in genes is more pronounced for co-directional than head-on interactions, consistent with literature evidence that replication initiation is enhanced in promotors of highly transcribed genes [36, 54], and we saw no convincing evidence of a TrAEL-seq enrichment at the 3′ end of genes in the head-on orientation that would indicate frequent collisions or break formation (Supplementary Fig. S4B).

Gene density is highest close to Initiation Zones in the human genome, so interactions with transcription may explain the difference in replication fork processivity around Initiation Zones as recently proposed [18]. We therefore re-assessed the relationship between TrAEL-seq read count and distance from Initiation Zones excluding data from highly transcribed regions based on PRO-seq read density [53], since TrAEL-seq read counts performed exclusively on less transcribed regions should be less affected by replication-transcription interactions. For this, we first generated a high resolution DLD-1 TrAEL-seq dataset by pooling technical replicates to obtain 49 million reads after deduplication, ∼4× our normal sequencing depth (10–20 million after deduplication). We then repeated the analysis of TrAEL-seq read density versus distance from Initiation Zone, for which read count is tallied in 50 kb windows across the genome, then average read counts are determined for windows lying at increasing distances from Initiation Zones (0–100 kb, 100–200 kb, etc). Although highly expressed genes are concentrated near Initiation Zones, more than half of the ∼9000 50 kb windows close to an Initiation Zones do not contain a highly expressed gene, so we were able to generate a plot excluding all the 50kb windows that contain a highly expressed gene (in the top quartile for PRO-seq read count). This had almost no effect on the profile (Fig. 4B), so we further excluded probes overlapping the next two quartiles; importantly, even when only including regions in the bottom quartile for transcription (where transcription is essentially undetectable by PRO-seq) the TrAEL-seq read density profile was identical to the complete TrAEL-seq dataset (Fig. 4B). Furthermore, an equivalent analysis in hESC showed that exclusion of transcribed regions increased the average TrAEL-seq read enrichment around Initiation Zones (Fig. 4B, right) [55]. We conclude that the variation in replication fork speed with distance from Initiation Zone measured by TrAEL-seq and other methods cannot be caused by interactions with transcription.

This observation contrasts with the report that treatment with the transcription inhibitor DRB accelerates replication fork speed such that fork speed becomes uniform [18]. Consistent with that finding, treatment of DLD-1 cells with DRB prevented the reduction in TrAEL-seq read density with distance from Initiation Zones indicating uniform fork speed, though if anything fork speed decreased with distance from origins (Fig. 4C). We also tested triptolide as a mechanistically distinct transcriptional inhibitor which showed an intermediate result as forks accelerated but only much further (1 Mb+) from defined Initiation Zones (Fig. 4C). However, these changes occurred without a complete loss of replication–transcription interactions, and triptolide had a stronger effect than DRB despite having a lesser effect on TrAEL-seq read distribution (Fig. 4D). These results are consistent with transcription inhibition affecting replication fork processivity but not directly through replication-transcription conflicts, and would be more consistent with an indirect effect mediated by inhibiting transcription of required factors such as cyclins [56].

Alternatively, it has long been known that replication fork speed is inversely proportional to the number of replication origins that fire, which can be attributed to limiting availability of replication factors (including but not limited to dNTPs) [19, 57–59], and we suggest that the same balance limits replication fork speed throughout S-phase. This hypothesis predicts that raising or lowering the number of origins that fire at early in S-phase should affect replication fork processivity and therefore alter TrAEL-seq read density. To test this, we first inhibited ATR to increase origin firing using Ceralasertib (AZD6738) [60–62], which increased origin firing detectable by TrAEL-seq (2475 and 2281 Initiation Zones detected in control samples by OKseqHMM versus 2752 and 2922 in Ceralasertib treated samples). As predicted this resulted in higher TrAEL-seq read density around replication Initiation Zones and a sharper decrease with distance (Fig. 4E). Conversely, to decrease origin firing at the start of S-phase we used CDK4/6 inhibition by palbociclib [63, 64], working in PC9 cells as DLD-1 cells are insensitive to CDK4/6 inhibitors and using a low dose of palbociclib (16 nM) that has no impact on proliferation. In this case, the slope of the TrAEL-seq signal was decreased on palbociclib treatment showing less change in fork processivity with distance from origins (Fig. 4F), though it should be noted that the small reductions in Initiation Zone firing efficiency expected from this mild treatment cannot be detected by TrAEL-seq.

These results demonstrate that although replication forks move faster far from replication initiation zones, this cannot be attributed to transcription–replication conflicts even though interactions between these machineries are readily detected.

## Conclusion

Overall, TrAEL-seq provides a wealth of information on replication fork dynamics with high sample throughput and low cost—the method involves no complicated procedures and has proved very reliable in our hands. Importantly, the capability of TrAEL-seq to analyse unsynchronized, unlabelled cell populations both minimises cell culture requirements and allows complex systems to be studied.

Here we have leveraged large and varied datasets to assess the effect of transcription on replication forks during normal proliferation: we find that replication fork processivity increases in regions further from Initiation Zones that would be replicated later in S-phase, and that transcription locally reduces replication fork processivity. However, these two observations are unconnected and the effect of distance from Initiation Zone (or time in S-phase) on replication fork progression rate is not the result of interactions with transcription.

We suggest that replication fork speed increases during S-phase simply because the total amount of replication possible given the availability of replication factors is constant across S-phase but the number of active replication forks progressively decreases. The decrease in the number of forks occurs because most replication initiation happens in the first half of S-phase [44], inter-origin distances are mostly relatively small (<1 Mb), so most replication forks have already terminated by late S-phase. Few areas of the genome are > 1 Mb from a replication initiation zone, and when these regions are replicated less other replication forks are active, so competition for replication factors is low and the forks can proceed faster.

## Supplementary Material

gkag212_Supplemental_Files

## Data Availability

Datasets generated in this work have been deposited at GEO accessions GSE299123 and GSE314665. The following publicly available datasets were also used: TrAEL-seq: mouse B cells [GSE279992]; hESCs under original protocol [GSE154811]; IMR32 [GSE186122]; KM-12 and HCT116 [GSE253197]; mouse embryonic stem cells [GSE259364]; SW-48 SMARCAL1 [GSE279461]; yeast meiosis [GSE154811, GSE287130]. OK-seq: Mouse B cells [GSE116319]. PRO-seq: DLD-1 [GSM7009627]; hESC [GSM7431765]

## Notes

Present address: Altos Labs Cambridge Institute, Granta Park, CB21 6GP Cambridge, United Kingdom
