# Supplementary material for: Multiplexed TrAEL-seq captures DNA replication dynamics in mammalian cells
Source: Nucleic Acids Res. 2026 Mar 14;54(5):gkag212. doi: 10.1093/nar/gkag212 (PMC12988324; doi:10.1093/nar/gkag212)
Supplement: gkag212_Supplemental_Files [file gkag212_supplemental_files.zip › Kara et al Supplementary Figures 1-4.pdf]

Supplementary Figures

A

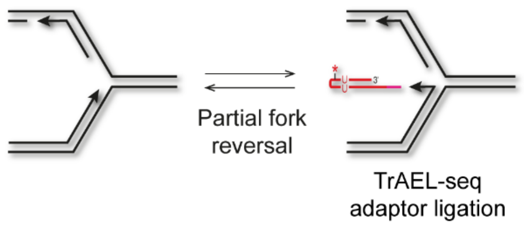

B

TrAEL adaptor	Index in adaptor	Index as read	Library quality
1	GACT	AGTC	Good
2	AGTC	GACT	Do not use
3	CAAG	CTTG	Good
4	TCGA	TCGA	Do not use
5	CCTT	AAGG	Good
6	GGAA	TTCC	Good
7	GCAC	GTGC	Good
8	TGGC	GCCA	Good
9	CATC	GATG	Medium

C

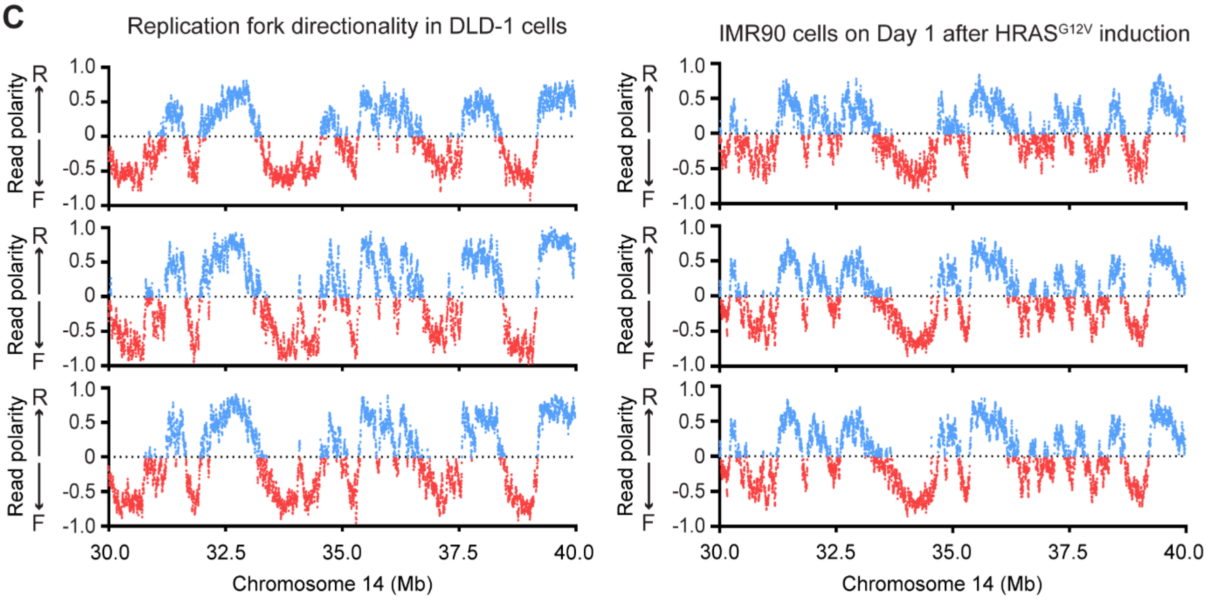

Figure S1: TrAEL-seq in mammalian cells

A: Schematic of replication fork conformational change to reveal leading strand 3' end. **B**: Table of multiplexing indexes tested, the chosen set of indexes for further use was 1, 3, 5, 6, 7, 8. **C**: Example RFD plots generated from 3 biological replicates each of DLD-1 cells and IMR90 cells at Day 1 of $HRAS^{G12V}$ induction.

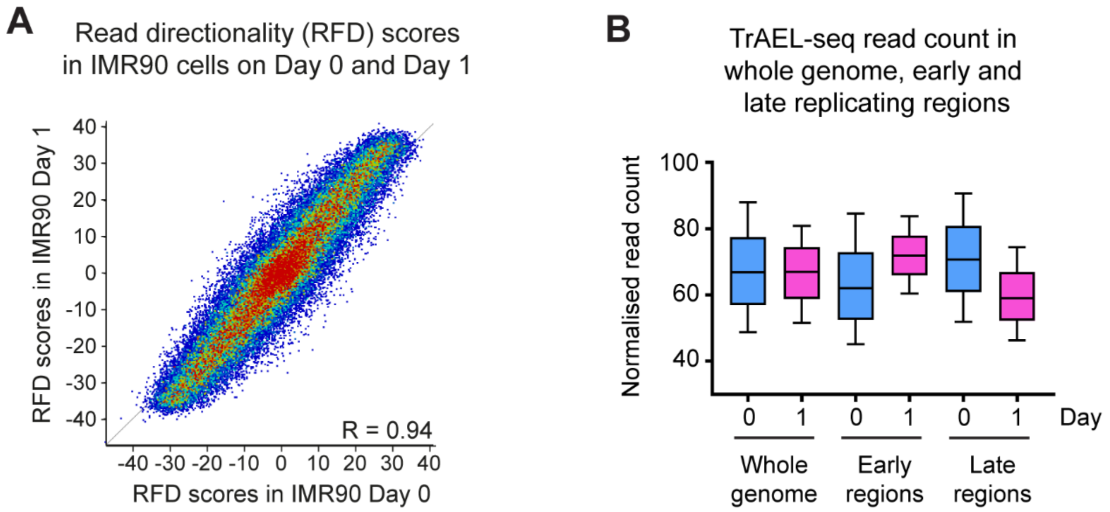

Figure S2: Supplement to multiplexed TrAEL-seq allows replication profiling in complex systems

A: Scatter plot of RFD values at all 50kb genomic regions (after filtering sites with aberrantly high or low read count) comparing IM90 cells on Day 0 and Day 1. **B**: Distribution of read counts in all 10kb windows across the genome (after filtering outliers) compared to windows overlapping early or late replicating genomic regions (based on Repli-Seq data for HCT116 (1)) for IMR90 cells on Day 0 and Day 1.

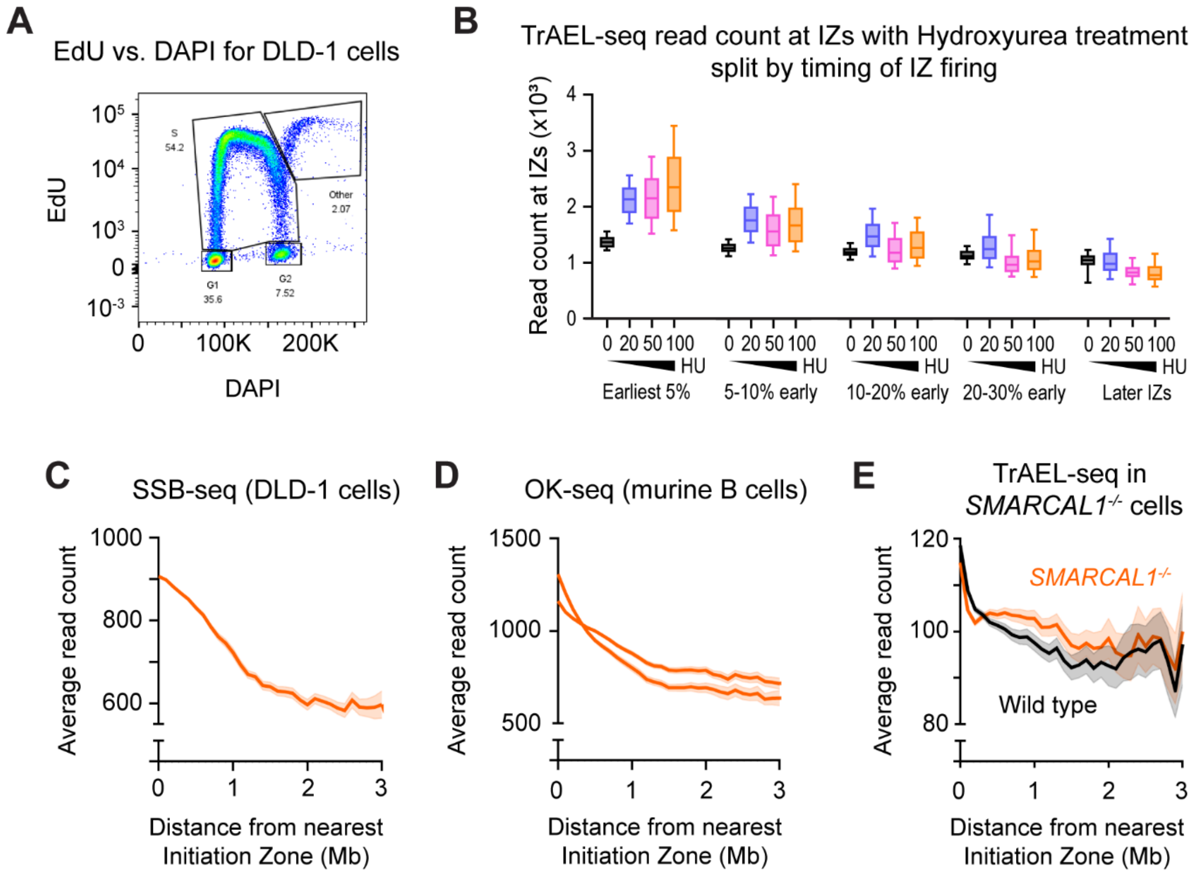

Figure S3: Supplement to TrAEL-seq profiling of variable replication fork density

A: Flow cytometry profile of DLD-1 cells after 3 hours incorporation of 10 μM EdU. **B**: TrAEL-seq read counts at IZs (centre of IZ ±100 kb), stratified based on replication timing (in HCT116 cells). **C**: Plot of total SSB-seq read count at increasing distance from replication Initiation Zones in DLD-1 cells. Solid line shows mean value, shaded band 95% confidence interval. Read counts were summed in 50kb windows spaced every 50 kb, with regions of altered copy number or aberrant read count removed. Distance was calculated from Initiation Zones determined using OKSeqHMM (2), and average read count determined in 100 kb windows of distance from the centre of initation zones. SSB-seq will detect both leading and lagging strand of replication forks as it is performed using an *in vitro* primer extension on total DNA with biotin-labelled dNTPs. **D**: Plot of total OK-seq read count from B cells using published data (3), solid lines show mean value, shaded band 95% confidence interval, analysed as in A. **E**: Plot of TrAEL-seq read count versus distance for SW48 cells versus SW48 *SMARCAL*$^{-/-}$ cells using data available at GSE279461. Solid line shows mean value, shaded band 95% confidence interval. Quantification as in C.

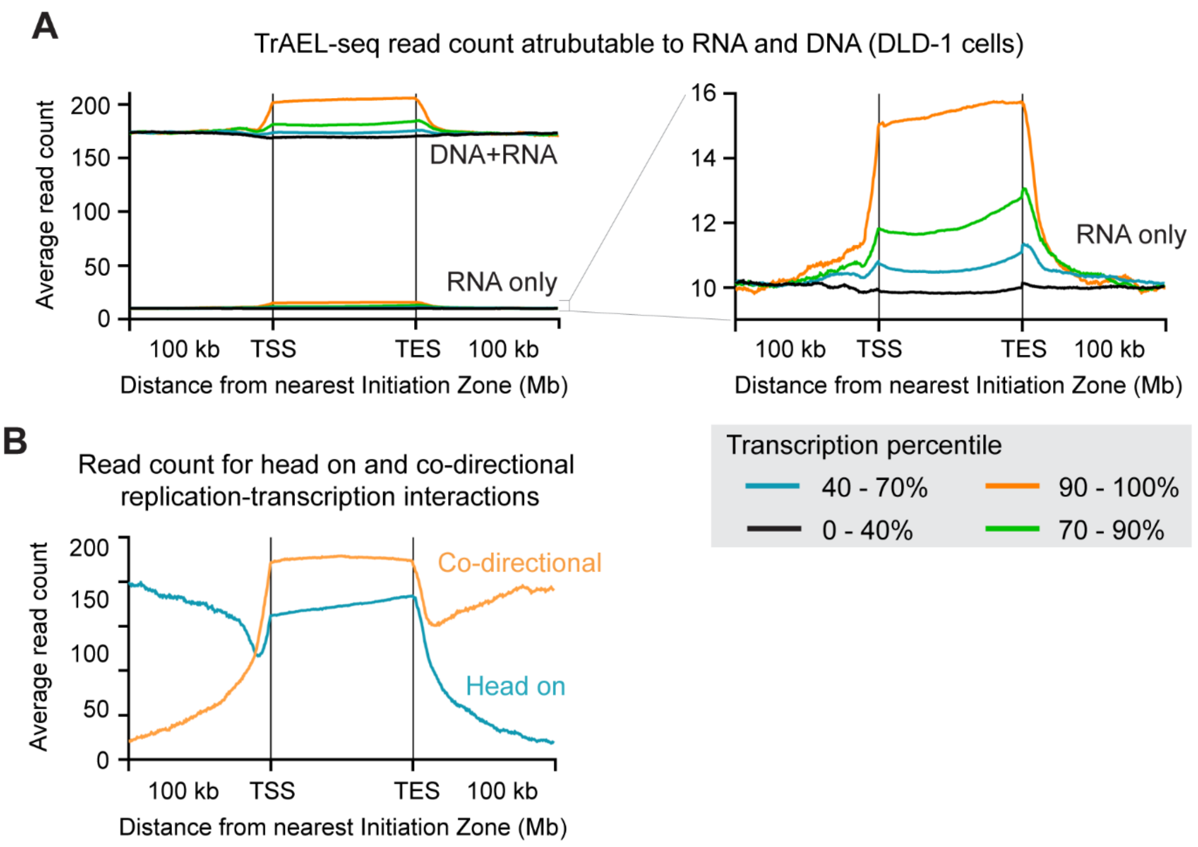

Figure S4: Supplement to causes of replication fork processivity differences across the genome.

A: Metaplot of TrAEL-seq read count in DLD-1 cells averaged across genes ±100 kb as in Figure 3C, showing both reads starting with T (which could derive from DNA or RNA) and separately the reads starting with other nucleotides (which are almost entirely RNA). The RNA count has been divided by 3 to compensate for this representing reads starting with 3 of the 4 nucleotides, the point of this comparison being to indicate the proportion of the reads in the DNA+RNA library that are likely to derive from RNA; even omitting this compensation, the proportion of the DNA+RNA signal arising through TrAEL-seq detection of nascent RNA remains a small part of the signal and cannot explain the observed enrichment of TrAEL-seq reads over transcribed regions. **B**: Metaplots as in Figure 4A, for highest expressed genes only divided into reads representing replication forks moving co-direction or head-on to transcribed regions.

1. Zhao, P.A., Sasaki, T. and Gilbert, D.M. (2020) High-resolution Repli-Seq defines the temporal choreography of initiation, elongation and termination of replication in mammalian cells. *Genome Biol*, **21**, 76.
2. Liu, Y., Wu, X., d'Aubenton-Carafa, Y., Thermes, C. and Chen, C.L. (2023) OKseqHMM: a genome-wide replication fork directionality analysis toolkit. *Nucleic Acids Res*, **51**, e22.
3. Tubbs, A., Sridharan, S., van Wietmarschen, N., Maman, Y., Callen, E., Stanlie, A., Wu, W., Wu, X., Day, A., Wong, N. *et al.* (2018) Dual Roles of Poly(dA:dT) Tracts in Replication Initiation and Fork Collapse. *Cell*, **174**, 1127-1142 e1119.
